# QSAR models for reproductive toxicity and endocrine disruption in regulatory use – a preliminary investigation†

**DOI:** 10.1080/10629360802550473

**Published:** 2008-12-05

**Authors:** G.E. Jensen, J.R. Niemelä, E.B. Wedebye, N.G. Nikolov

**Affiliations:** National Food Institute, Department of Toxicology and Risk Assessment, Technical University of Denmark, Søborg, Denmark

**Keywords:** QSAR, reproductive toxicity, advisory classifications, endocrine disruption

## Abstract

A special challenge in the new European Union chemicals legislation, Registration, Evaluation and Authorisation of Chemicals, will be the toxicological evaluation of chemicals for reproductive toxicity. Use of valid quantitative structure–activity relationships (QSARs) is a possibility under the new legislation. This article focuses on a screening exercise by use of our own and commercial QSAR models for identification of possible reproductive toxicants. Three QSAR models were used for reproductive toxicity for the endpoints teratogenic risk to humans (based on animal tests, clinical data and epidemiological human studies), dominant lethal effect in rodents (*in vivo*) and *Drosophila melanogaster* sex-linked recessive lethal effect. A structure set of 57,014 European Inventory of Existing Chemical Substances (EINECS) chemicals was screened. A total of 5240 EINECS chemicals, corresponding to 9.2%, were predicted as reproductive toxicants by one or more of the models. The chemicals predicted positive for reproductive toxicity will be submitted to the Danish Environmental Protection Agency as scientific input for a future updated advisory classification list with advisory classifications for concern for humans owing to possible developmental toxic effects: Xn (Harmful) and R63 (Possible risk of harm to the unborn child). The chemicals were also screened in three models for endocrine disruption.

## 1. Introduction

The need for prediction of the toxicity of untested chemicals is still increasing. The new regulation of chemicals in Europe (REACH – Registration, Evaluation and Authorisation of Chemicals) requires all chemicals manufactured in or imported into the European Union (EU) community in volumes greater than 1 tonne per year to be registered and undergo human health hazard assessment [1]. The tests for reproductive toxicity are among the most costly tests, requiring testing on vertebrate animals. It is estimated that approximately 30,000 chemicals will require registration [2] and that 30% of the total testing costs will be used for the development of toxicity studies [3]. However, it is at present not possible to fulfil all the REACH information demands by means of experimental testing only. Under the new legislation, predictions from valid quantitative structure–activity relationships (QSARs) may be used to fill data gaps.

In 2001, the Danish Environmental Protection Agency (EPA) made an advisory list for self-classification of dangerous substances [4] based on the QSAR work of our group (the ‘Danish QSAR group’). According to EU classification legislation [5], it is the duty of the manufacturers/importers to assess whether a substance they wish to introduce to the market should be classified. Often very little information is available on the danger posed to human beings and the environment for chemical substances on the European market [6]. The Danish advisory classification list included advisory classifications for substances without harmonised EU classifications (Annex I of Directive 67/548/EEC) for the endpoints acute oral toxicity, sensitization by skin contact, mutagenicity, carcinogenicity, and danger to aquatic environment. Since then, the Danish QSAR group has continued working in the field and many more QSAR models as well as improved software tools are now available. Current modelling systems include Multiple Computer Automated Structure Evaluation (MultiCASE), Oasis Database Manager, Leadscope, MDL and Meta. The QSAR model battery to be used for a possible future update of the advisory list includes at present 39 models. The aim of the investigation presented in this article is to set up a QSAR screening algorithm for reproductive toxicity for the parts of the effect, where we at this point have valid models. These do not cover all possible mechanisms of reproductive toxicity. Reproductive toxicity is not a clear-cut endpoint. Human reproduction is complex and so is reproductive toxicity. Reproductive toxicity may be evaluated on the basis of human data (cases and clusters, descriptive epidemiology and analytical epidemiology), animal experiments (generation experiments, teratogenicity experiments, behavioural teratogenicity experiments, sperm morphological tests and 28-days tests), as well as *in vitro* teratogenicity tests, where embryonic cells are harvested and exposed to the chemicals in question [7,8]. In our QSAR screening algorithm for reproductive toxicity, we included models for teratogenic risk and mutagenicity in germ cells: Rodent dominant lethal effect (*in vivo*) *and Drosophila melanogaster* sex-linked recessive lethal (SLRL) effect (*in vivo*). The dominant lethal test in rodents and the *Drosophila* SLRL test are initially meant for genotoxicity effects on germ cells, but the resulting effect is early embryonic deaths and lethal effect on offspring of females, respectively. Therefore, the endpoints are relevant for reproductive toxicity assessment.

As we have also developed a number of endocrine disruption (ED) models, we ran the models on the predicted positive reproductive toxicants to see whether these effects seemed like possible mechanisms of reproductive toxicity. Three models for ED were used, predicting estrogen α receptor binding, estrogen reporter gene activation and androgen receptor antagonism.

## 2. Materials and methods

### 2.1 Toxicological data

Data for the training sets for the models were obtained from the literature and from our own experimental tests. A commercial MultiCASE training set constitutes the basis of one model. The numbers of chemicals in the training sets of the QSAR models are given in Table 1.

**Table 1:** Training set information for models for reproductive toxicity and endocrine disruption.

	Total (n)	Positive (n)	Negative (n)
Reproductive toxicity			
Teratogenic risk	323	130	193
Rodent dominant lethal	191	78	113
*Drosophila* m. SLRLU	377	190	187
Endocrine disruption			
Estrogen α receptor binding	595	284	311
Estrogen reporter gene	481	195	286
Androgen receptor antagonism	523	242	281

#### 2.1.1 Teratogenic risk (*in vivo*)

The model is the MultiCASE commercial model A49 [9,10]. The training set is composed of data taken from the Teratogen Information System (TERIS) and a compilation in which the US Food and Drug Administration (FDA) definitions were used to quantify risk of developmental toxicity from drugs used during pregnancy. The training set consists of clinical, epidemiologic and animal data. Many biological mechanisms are involved in the effects.

#### 2.1.2 Dominant lethal effect in rodents (*in vivo*)

The training set is comprised of data from Green et al. [11] and other references. In the experimental method, mice and rats are used. Male animals are treated acutely, sub-acutely or over the entire period of spermatogenesis. Treated animals are mated according to an experimental scheme. Females are usually killed at 14 days of pregnancy and implantations examined. The category of early embryonic deaths is the most significant index of dominant lethality and as such used as endpoint. The test identifies chromosomal aberrations as well as point mutations in spermatocytes.

#### 2.1.3 Drosophila melanogaster SLRL effect (*in vivo*)

The training set consists of data from Lee et al. [12]. In the experimental method, *D. melanogaster* males and females are used. Males are treated with the test substance and mated individually to virgin females. The test detects the occurrence of mutations, point mutations and small deletions, in the germ line of the insect. The mutations are phenotypically expressed in males carrying the mutant gene. When the mutation is lethal in the hemizygous condition, its presence is inferred from the absence of one class of male offspring out of the two that are normally produced by a heterozygous female.

#### 2.1.4 Estrogen α receptor binding (*in vitro*)

The training set uses data from METI [13]. In the experimental method, human estrogen receptor produced from *Escherichia coli* was used. The chemical substance is added to a system where RI-labelled estrogen as reference hormone binds to the human estrogen receptor. The chemical concentration that inhibits 50% of the binding of the reference hormone to the receptor is measured and defined as IC_50_. As endpoint units, relative binding affinity (RBA) between the IC_50_ values of the chemical and a natural hormone (E2, etc.) when the IC_50_ concentration of natural hormone is set at 100 was used.

#### 2.1.5 Estrogen reporter gene (*in vitro*)

The training set was also derived from METI [13]. In the experimental method, the estrogen effect of chemicals was measured as an increase of the luminescence response induced by the synthetic estrogene E2 in harvested MCF-7 cells. As endpoint units the increase in luminescence response was used as active/not active intersection point. The test identifies chemicals, which have influence on estrogen receptor binding and transactivation of the receptor followed by estrogen-dependent gene expression.

#### 2.1.6 Androgen receptor antagonism (*in vitro*)

The training set contains data from own experimental testing and data from the literature [14]. In our experimental method, the androgen receptor antagonism of chemicals was measured as the inhibition of the luminescence response induced by the synthetic androgen R1881 in harvested Chinese Hamster Ovary (CHO) cells. As endpoint units, inhibition of luminescence response as active/not active intersection point was used. The activity is observed as an inhibition of the progress of androgen receptor binding and transactivation of the receptor followed by gene expression visualized by enzyme response.

### 2.2 Software and hardware

The MultiCASE system [9] was used in this investigation to compile modules for specific toxicological endpoints. Calculations were performed on a standard PC under Windows operative system. The results of the experimental studies, which went into the training sets of the models, were converted to MultiCASE numerical activity units [4,14].

MultiCASE uses simplified molecular input line entry system (SMILES) codes to enter chemicals. The program is a fragment-based statistical model system that aims to discover fragment combinations, which are relevant to the observed effect. Biophores are structural alerts that appear mostly in active molecules and therefore may be responsible for the observed activity. MultiCASE starts by identifying the statistically most significant substructure existing within a training set. This fragment, labelled the top biophore, is seen as responsible for the activity of the largest possible number of active molecules. The molecules containing this biophore are then removed from the database and the remaining ones are submitted to a new analysis leading to the identification of the next biophore. This procedure is repeated until either the activity of all the molecules in the training set have been accounted for or no additional statistically significant substructure can be found. The chemicals containing the same biophore are grouped together. For each set of molecules containing a specific biophore, MultiCASE identifies additional parameters, deemed modulators. These modulators may be structural fragments or chemical properties (e.g. log P, HOMO/LUMO energies, or water solubility) that can either enhance or inhibit the activity of the chemicals containing the biophore. The relevant modulators are then used to derive a QSAR restricted to the chemicals containing the biophore. MultiCASE also looks at inactives in the training set to identify deactivating fragments, deemed biophobes.

### 2.3 Statistical validation

The QSAR models were validated by cross-validation. Robust cross-validation was chosen as the procedure required to test the predictivity [15]. While drawbacks of cross-validation exist [16,17], much of the criticisms are directed towards the simpler leave-one-out cross-validation [16]. In this paper we use the more stable leave-many-out cross-validation by leaving out random pos/neg balanced sets of 50% of the chemicals, repeated 10 times. Cooper statistics from the cross-validations are summarized in Table 2. Concordances for the models ranged from 75.9 to 82.5%, with sensitivities ranging from 41.3% to 77.3% and specificities ranging from 84.2 to 95.2%. The androgen receptor antagonism model was also externally validated with somewhat higher concordance result than from the 50% cross-validation.

**Table 2:** Cross-validation results for the QSAR models.

	Sensitivity (%)	Specificity (%)	Concordance (%)
Reproductive toxicity			
Teratogenic risk	50.2	91.3	79.3
Rodent dominant lethal	41.3	95.2	75.9
*Drosophila* m. SLRL	73.9	88.1	81.6
Endocrine disruption			
Estrogen αreceptor binding	77.3	86.5	82.5
Estrogen reporter gene	46.4	94.9	80.9
Androgen receptor antagonism*	64.4	84.2	76.1

Note: The androgen receptor antagonism model was also validated by prospective external validation, where a validation set was chosen after the model had been constructed by randomly choosing test chemicals within the applicability domain. The chemicals were double blinded and tested in our laboratory. The external validation with 102 chemicals resulted in a sensitivity of 57%, a specificity of 98%, and a concordance of 92% of the model [14].

### 2.4 Applicability domain

During the prediction process for a given substance, MultiCASE may provide warnings due to the presence of fragments not present in the training set and not covered by the model, or the presence of inactivating fragments associated with an active prediction (or the opposite). In this investigation, any MultiCASE warning was considered being an indication that the molecule was outside the model domain. This maximizes the concordance but lowers the sensitivity. Sensitivities of the applied models range between 41 and 77%.

For each model the applicability domain was expressed as the percentage of predictions without warnings out of a structure set of 57,014 discrete organic EINECS chemicals in the Danish QSAR database [14]. The domains of the QSAR models are shown in Table 3. The domains of the models used to predict reproductive toxicity ranged between 43 and 56%. Thus, the models used can predict about half of the screened EINECS chemicals.

**Table 3:** Domains of the models within 57,014 EINECS substances.

	Domain (%)
Reproductive toxicity	
Teratogenic risk	43
Rodent dominant lethal	48
*Drosophila* m. SLRL	56
Endocrine disruption	
Estrogen α receptor binding	52
Estrogen reporter gene	61
Androgen receptor antagonism	56

### 2.5 Mechanistic interpretation

The MultiCASE does not have any preconceived knowledge of molecular events that explain the activity of a molecule. However, many of the resulting predictions have modes of action that are obvious to persons with expert knowledge for the endpoint in question. Other groups represent mechanisms of action, which require further examination to try and elucidate their toxicological modes of action. Provided that the model is sufficiently predictive, we consider the additional ability to suggest new hypotheses based on chemical substructures to be an extremely desirable feature, rather than the opposite.

## 3. Results and discussion

### 3.1 Screening algorithm for reproductive toxicity

**Figure 1:**
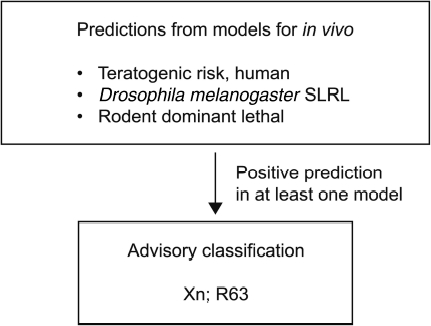
Algorithm for reproductive toxicity screening.

The models for teratogenic risk, rodent dominant lethal effect and *D. melanogaster* SLRL were included in the algorithm and applied in the screening of the EINECS structure set of 57,014 chemicals. Chemicals were considered predicted positive for reproductive toxicity if a positive prediction was obtained in any of the models within the applicability domain (Figure 1). A total of 5240 chemicals were predicted positive by this procedure, corresponding to 9.2% of the 57,014 EINECS chemicals. Of these, 2331 (44% of the 5240) were identified by the teratogenic risk model and the rest were identified on the basis of the genotoxicity models exclusively. Table 4 lists the numbers of chemicals predicted positive by the individual models. A number of chemicals were identified by both the teratogenic risk model and one or both of the genotoxicity models (Table 5). Out of the 2,331 chemicals predicted positive by the teratogenic risk model, 349 chemicals (15%) were also predicted positive by one or both of the genotoxicity models. For these chemicals the predicted reproductive toxicity effect may be due to mutation in germ cells or mutation may be an active mechanism prior to the teratogenic effect. In many cases, a toxicological threshold is assumed to exist for reproductive toxicity. With mutagenic chemicals this may not be the case, and they may therefore be of even greater concern.

**Table 4:** Prediction of 57,014 EINECS chemicals for reproductive toxicity.

QSAR models	Chemicals (n)	Chemicals within domain (n)	Positive chemicals (n)	Positive chemicals (%)
Teratogenic risk	57,014	24,516	2331	9.5
Rodent dominant lethal	57,014	27,366	1906	7.0
*Drosophila* m. SLRL	57,014	31,927	1668	5.2
Reproductive toxicity, total^a^	57,014		5240	9.2

a If positive in at least one model.

**Table 5:** Prediction of 57,014 EINECS chemicals for teratogenic risk, genotoxic relation.

QSAR models	Unit	Positive chemicals
Teratogenic risk	n	2331
Teratogenic risk and rodent dominant lethal or *Drosophila* m. SLRL	n	349
Genotoxic relation	%	15

### 3.2 Advisory classifications for reproductive toxicity

Reproductive toxicants are according to the EU classification criteria classified into three categories with associated hazard symbol and risk sentences. The categories reflect the degree of documentation for the effect rather than the potency or seriousness of the effect [5,18,19]. Annex V of the EU classification directive lists methods for the determination of toxicity. The teratogenicity test, the rodent dominant lethal test and the *Drosophila melanogaster* SLRL test are included on the list. The EU classification criteria include the possibility of using expert judgements as well as conclusions based on structural analogies. The list with positive screening findings for 5240 chemicals with the suggested classification for reproductive toxicity in category 3: Xn (Harmful) and R63 (Possible risk of harm to the unborn child) will be submitted to the Danish EPA as scientific input to a future update of the advisory classification list. Further modifications of the screening procedure may be relevant for that purpose.

### 3.3 Possible ED mechanism in reproductive toxicity

According to the literature, endocrine disrupters may lead to reproductive disorders in adults, tumour developments in adults and offspring, deterioration of genital organ development in offspring and developmental neurotoxicity in offspring. The mechanisms involved may be altered hormonal function through receptor recognition/binding, altered hormone biosynthesis, altered hormone storage and/or release, altered hormone transport and clearance, altered post-receptor activation and others [20]. As we have developed a number of models for ED (*in vitro*) endpoints, we undertook an evaluation to see how much of the predicted reproductive effect might be due to ED mechanisms. The models covered mechanisms for androgen receptor antagonism, estrogen α receptor binding, and activation of the estrogen receptor. Thus the three endpoints for ED used in this investigation only cover part of the possible ED mechanisms in reproduction. The 5240 chemicals predicted positive for reproductive toxicity were run in the three models. Positive predictions were found for 5.3% in the androgen receptor antagonism model, 4.7% in the estrogen α receptor binding model, and 3.3% in the estrogen reporter gene model (Table 6). According to the models, it seems that ED is not the most common mechanism for reproduction toxicity in EINECS chemicals. The three *in vitro* ED tests for androgen receptor antagonism, estrogen α receptor binding, and activation of the estrogen receptor are extremely sensitive. While they may provide important mechanistic information, it is not suggested that classification be based on model predictions of them.

**Table 6:** Prediction of 57,014 EINECS chemicals for reproductive toxicity, endocrine disruption relation

QSAR models	Positive chemicals (n)	Positive chemicals (%)
Reproductive toxicity	5240	
Teratogenic risk or rodent dominant lethal or *Drosophila* m. SLRL		
Reproductive toxicity and androgen receptor antagonism	278	5.3
Reproductive toxicity and estrogen reporter gene	172	3.3
Reproductive toxicity and estrogen α receptor binding	244	4.7

### 3.4 Examples of possible mechanisms in the models

**Figure 2:**
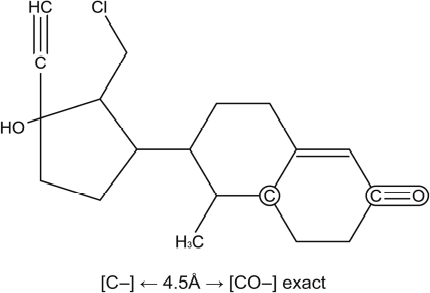
Chemical no. 1 predicted by the teratogenic risk model

**Figure 3:**
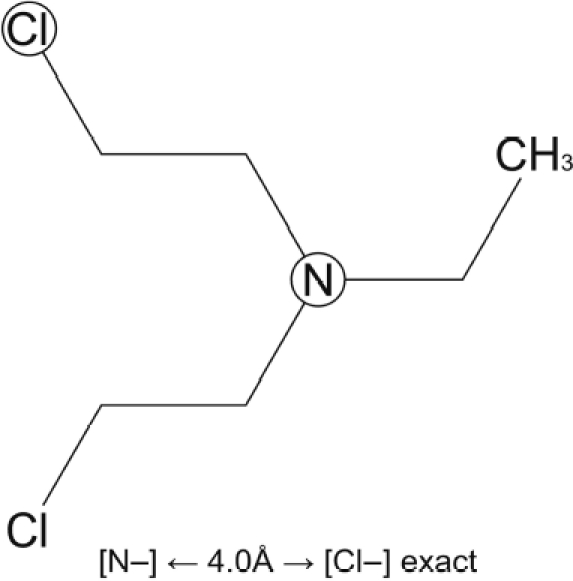
Chemical no. 2 predicted by the rodent dominant lethal model.

Two chemicals (chemical no. 1 and chemical no. 2) were used to illustrate the possible mechanisms involved in the reproductive toxicity effect. Both chemicals were predicted positive by the teratogenic risk model. The MultiCASE program identifies chemical substructures (biophores) linked to active molecules. The structures of chemical no. 1 and chemical no. 2 with illustration of identified biophores are given in Figures 2 and 3.

Chemical no. 1 had in addition to the positive prediction in the teratogenic risk model, also a positive prediction in the androgen receptor antagonism model. However, it gave negative predictions in the other ED models and in the two models for genotoxicity. Thus, ED by androgen receptor antagonism may be involved in the reproductive toxicity effect of chemical no.1. In the teratogenic model, the distance fragment [C–] ←4.5 Å → [CO–] was identified as a biophore. Within the 15 chemicals forming basis for the biophore three distinct classes came out; progestogens, barbituates and warfarin derivatives. All three groups are well-known reproductive toxicants.

Chemical no. 2 had in addition to the positive teratogenic risk prediction, also positive predictions in the two models for mutation in germ cells. However, it gave negative predictions in the ED models. Thus, the reproductive toxicity effect of chemical no. 2 may be due to a genotoxic mechanism. In the rodent dominant lethal model the distance biophore [N–] ← 4.0 Å → [Cl–] and the biophore Cl–CH_2_–CH_2_ were identified. Basically all the chemicals in the training set forming basis for these biophores appeared to be alkylating agents, i.e. can damage DNA.

### 3.5 Discussion

Annex I of the EU classification directive today contains harmonized EU classifications covering about 8000 chemicals out of the EINECS list with 100,204 entries. Of the 8000 chemicals covered by Annex I, less than 200 chemicals or group entries have classifications for reproductive effects (R60, R61, R62 or R63), possibly partly due to limited testing for reproductive toxicity. With the new EU REACH legislation, it is anticipated that there will be a significant requirement for reproductive toxicity testing. It is well known that these tests use many animals [3] and have significant economic cost. This has stimulated interest in the area of alternatives for reproductive toxicity testing, including the use of *in silico* approaches. Human reproductive toxicity is a complex endpoint with many mechanisms of action. We have looked at a few of them in the investigation presented in this paper. There are many other recognized and well-known mechanisms/chemical groups responsible for reproductive toxicity, like, e.g. cholesterol modulators (statins), thyroid modulators, angiogenesis inhibitors, and small organic acids. In the future, we hope to be able to model some of these additional endpoints.

## 4. Conclusion

Quantitative structure–activity relationship models for teratogenic risk, rodent dominant lethal effect and *D. melanogaster* SLRL effects were used to predict reproductive toxicity. A total of 5240 EINECS chemicals corresponding to 9.2% of the available and screened 57,014 EINECS structures were predicted as reproductive toxicants. ED *in vitro* for three individual ED endpoints explained 3–5% of the reproductive toxicity effect. The positive QSAR screening results for reproductive toxicity will be submitted to the Danish EPA for possible inclusion on a future update of the advisory classification list with the suggested classification for reproductive toxicity: X_n_ (Harmful) and R63 (Possible risk of harm to the unborn child).
